# The Great Escape: Viral Strategies to Counter BST-2/Tetherin

**DOI:** 10.1371/journal.ppat.1000913

**Published:** 2010-05-13

**Authors:** Janet L. Douglas, Jean K. Gustin, Kasinath Viswanathan, Mandana Mansouri, Ashlee V. Moses, Klaus Früh

**Affiliations:** Vaccine and Gene Therapy Institute, Oregon Health & Science University, Beaverton, Oregon, United States of America; The Fox Chase Cancer Center, United States of America

## Abstract

The interferon-induced BST-2 protein has the unique ability to restrict the egress of HIV-1, Kaposi's sarcoma–associated herpesvirus (KSHV), Ebola virus, and other enveloped viruses. The observation that virions remain attached to the surface of BST-2-expressing cells led to the renaming of BST-2 as “tetherin”. However, viral proteins such as HIV-1 Vpu, simian immunodeficiency virus Nef, and KSHV K5 counteract BST-2, thereby allowing mature virions to readily escape from infected cells. Since the anti-viral function of BST-2 was discovered, there has been an explosion of research into several aspects of this intriguing interplay between host and virus. This review focuses on recent work addressing the molecular mechanisms involved in BST-2 restriction of viral egress and the species-specific countermeasures employed by various viruses.

## Introduction

BST-2 (CD317/HM1.24) was initially identified by two independent groups searching for novel surface markers of terminally differentiated normal and neoplastic B cells [1], [2]. In a proteomics screen, our group subsequently identified BST-2 as a novel target for the viral ubiquitin-ligase K5 of Kaposi's sarcoma–associated herpesvirus (KSHV) [3]. However, the function of BST-2 remained unknown until it was identified as an intrinsic anti-viral factor that restricts the egress of HIV-1 by tethering mature virions to the host cell surface [4]. Coincident with this discovery, BST-2 was identified as a target of the HIV-1 accessory protein Vpu, providing a plausible mechanism for the well-established, but ill-defined, virus release function of Vpu [4]. Work by other investigators showing that Vpu downregulates BST-2 from the cell surface [3], [5] suggested a mechanism for Vpu antagonism of BST-2. These discoveries have stimulated an active area of research that explores several intriguing aspects of BST-2 function, including its role as a general inhibitor of enveloped virus release, the mechanisms underlying its neutralization by viral immunomodulators, and the possibility that additional activities for this enigmatic protein remain to be identified. In addition to providing a critical overview of recent discoveries in the field, the intent of this review is to summarize the history of BST-2, its anti-viral activities, and potential modes of action. We focus primarily on human BST-2 and HIV-1 to describe the molecular characteristics of BST-2, countermeasures employed by HIV-1 Vpu, and the genetic and mechanistic aspects of the host–virus interaction. To put the significance of BST-2/HIV-1 into a larger perspective, we also address species specificity and discuss other viruses restricted by BST-2, and the means, if any, utilized by these viruses to overcome BST-2. While much remains to be clarified regarding the nature and significance of BST-2 function, its role as an intrinsic mediator of anti-viral resistance provides unique insight into the complexity of host–virus relationships and reminds us of the potential to exploit these relationships for therapeutic benefit.

## Molecular Characteristics of BST-2

### Membrane Topology of BST-2

**Figure 1:**
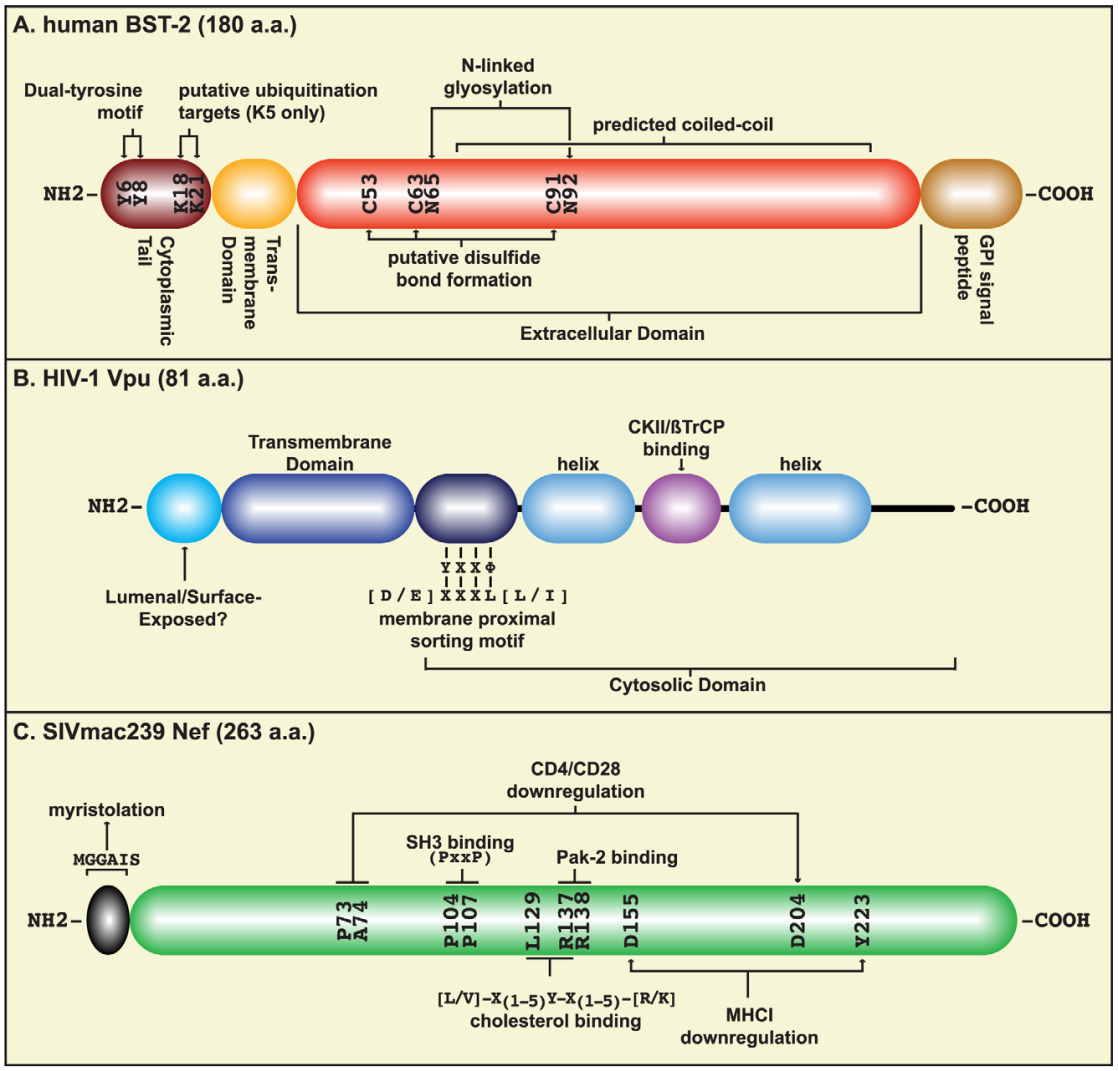
Host and viral factors involved in virion release. Schematics of human BST-2 (A), HIV-1 Vpu (B), and SIVmac239 Nef (C) proteins with salient features indicated. The coiled-coil domain of BST-2 was predicted using PCOILS (http://toolkit.tuebingen.mpg.de/pcoils) [58].

Human, rat, and mouse BST-2 have been independently identified and subsequently cloned by several groups [2], [6]–[8]. This work and that of others [9] revealed that *bst-2* encodes a 20-kDa, single pass, type II glycosylated membrane protein that localizes to lipid rafts via its COOH-terminal glycosylphosphatidylinisotol (GPI) anchor (Figure 1A). While BST-2 migrates as a heterogenous smear of approximately 30–36k Da in reducing SDS-PAGE, the protein migrates as a larger dimer under non-reducing conditions, presumably due to the formation of disulfide bonds among the three conserved cysteine residues in the extracellular domain. Among known proteins, this topology is relatively unique, as it has only been observed for one variant of the prion protein [10].

### Sub-Cellular Localization and Transport of BST-2

BST-2 localizes not only to the plasma membrane but also to internal membranes, particularly the trans-Golgi network (TGN) and recycling endosomes [6]. Unlike other GPI-anchored proteins, BST-2 is endocytosed from the cell surface in a clathrin-dependent manner. This appears to depend upon an interaction between an AP-2 subunit and a non-canonical, dual tyrosine motif within the BST-2 cytosolic domain [9], [11] (Figure 1A). Recent studies also show that BST-2 expressed at the apical surface of polarized epithelial cells is linked to the actin cytoskeleton through a series of ezrin-radixin-moesin (ERM)-binding and adapter proteins: RICH2, EBP50, and ezrin. Furthermore, siRNA knockdown of BST-2 in these cells resulted in a re-organization of the actin cytoskeleton in a Rac- and Rho-dependent manner [12]. While the implications of these interactions for the anti-viral function of BST-2 have not yet been evaluated, BST-2 appears to locate to subcellular sites frequently used for viral egress.

### Transcriptional Regulation of BST-2

Within the *bst-2* promoter region is a tandem repeat containing interferon (IFN) response elements and three STAT3 binding sites that are activated in response to interleukin (IL)-6 [7]. Indeed, BST-2 is upregulated in most mouse and human cell types upon type I and type II IFN treatment [4], [8], consistent with an evolutionarily conserved innate immune function. Interestingly, BST-2 can also inhibit the production of IFN and pro-inflammatory cytokines, such as IL-6 and tumor necrosis factor (TNF)-α by human plasmacytoid dendritic cells (pDCs) [13]. This inhibition is accomplished by BST-2 binding to the orphan receptor immunoglobulin-like transcript 7 (ILT7), which is expressed exclusively on pDCs. This interaction establishes a negative feedback loop in which IFN-induced BST-2 binds to the ILT7-FcεRIγ complex, thereby signaling the inhibition of IFN and proinflammatory cytokines [13]. In addition to the well-studied anti-viral function described below, BST-2 might also play a role in regulating innate immune cells.

## Mechanisms of BST-2 Anti-Viral Restriction and Vpu Countermeasures

The HIV-1 accessory protein Vpu is a small NH2-terminally anchored TM protein that mediates the degradation of CD4 [14] via interaction with the beta transducin repeat-containing protein (βTrCP), a subunit of the Skp1-Cullin-F-box (SCF) E3 ubiquitin ligase complex [15] (Figure 1B). In addition, Vpu enhances the release of progeny virions from certain cell types (“Vpu responsive” cells), a phenomenon that was discovered 20 years ago [16]. However, the mechanism of this enhancement remained obscure. The recent identification of BST-2 as a putative viral restriction factor subject to Vpu antagonism has answered a long-standing question regarding Vpu's virion release function. However, it has also stimulated many intriguing new questions about the evolution and function of both of these proteins.

### Evidence for a BST-2 Anti-Viral Tethering Function

Electron micrographs of Vpu-responsive cells infected with ΔVpu HIV-1 reveal the presence of viral particles accumulated at the cell surface in what appear to be tethered chains [16]. In two seminal papers it was shown that the expression of BST-2 confers the Vpu-responsive phenotype, and that in cells lacking BST-2 expression, there is a marked reduction in “tethered” ΔVpu virions [4], [5]. To reflect this unique activity, BST-2 was renamed “tetherin” [4]. Tethered, cell-associated virions appear to be fully mature, based on the presence of both electron dense cores and the functional reverse transcriptase activity of particles that have been physically dislodged from the infected cell surface [16]. The virions can also be released by protease treatment, which Neil et al. present as evidence for a protein-based tether, as opposed to a budding defect that prevents membrane separation [4], [17]. However, this protease sensitivity does not rule out a potential role for other host proteins besides BST-2 in restricting virion release or alternative hypotheses to tethering as the mechanism of viral restriction. A recent report has identified BCA2 as a BST-2-interacting factor, which is thought to supplement the BST-2 viral restriction by enhancing the internalization and degradation of tethered virions from the cell surface [18]. Because BST-2 can restrict a large number of enveloped viruses (see Table 1), it is unlikely that it interacts with a specific viral protein to induce tethering. Neil et al. hypothesized that because BST-2 forms dimers and higher order multimers, perhaps BST-2 is incorporated into virions, thereby allowing for tethering between virus- and cell-associated BST-2 [4]. Perez et al. tested this hypothesis and found that they could only detect BST-2 in ΔVpu HIV-1 particles when BST-2 was functionally inactivated via deletion of either its transmembrane (TM) domain or GPI anchor [19]. Wild-type BST-2 could only be detected in over-expressed Gag viral-like particles (VLPs). Interestingly, only the ΔTM mutant was incorporated into wild-type HIV-1 virions, suggesting that Vpu limits BST-2 incorporation into viral particles via the TM domain. Several other reports have confirmed the incorporation of BST-2 into HIV virions, although discrepancies remain. For example, Hammonds et al. [20] were able to detect IFN-induced BST-2 in ΔVpu virions, but not wild-type HIV virions, while Fitzpatrick et al. [21] and Habermann et al. [22] detected endogenous BST-2 in both wild-type and ΔVpu HIV. In contrast to these studies, Miyagi et al. were able to detect endogenous BST-2 in ΔVpu, but not wild-type HIV virions released via vortexing from infected cells [23]. However, they also detected BST-2 in control preparations of vesicles isolated from uninfected cells, and therefore concluded that BST-2 is not specifically incorporated into viral particles. Neil et al. [4] went on to hypothesize that if BST-2 were incorporated into viral particles, a tethering mechanism might depend upon homo-dimeric/oligomeric interactions between cell- and virus-associated BST-2 molecules. This has been tested by several groups. Treatment of cell surface-tethered HIV and Ebola VLPs [24] or wild-type HIV [21] with reducing agents did not induce particle release, suggesting that tethering does not involve disulfide linkage of BST-2 dimers or oligomers. Similarly, treatment of tethered virions with the GPI anchor-cleaving enzyme Pi-PLC did not effectively release the virions [21]. Thus, while it is now clearly established that “Vpu-responsiveness” is caused by BST-2, additional studies are required to further elucidate the BST-2-dependent tethering mechanism and to determine whether there is a functional role for virion-associated BST-2.

**Table 1:** Viruses Restricted by BST-2 and Their Countermeasures.

Virus	BST-2 Antagonist	Mechanism	Species Specificity of Antagonist	Reference
HIV-1	Vpu	Cell surface downregulation/degradation	Yes	See Table 3
SIVmus/gsn/mon	Vpu	Presumably same as HIV-1 Vpu	Yes	[44], [48], [49]
SIVcpz/gor	Nef (although it expresses Vpu)	?	Yes	[48], [49]
SIVmac	Nef	?	Yes	[41], [55]
SIVagm	Nef	?	Yes	[55]
SIVagm	Env	cell surface downregulation/sequestration	No	[56]
SIVagm	None	“Not needed”	N/A	[44]
HIV-2	Env	Cell surface downregulation/not degradation	No	[30], [41], [54]
Other lentiviruses (EIAV, FIV); other retroviruses (RSV, MPMV, HTLV-1,PFV)	? not evaluated	N/A	N/A	[59]
Filoviruses (Ebola, Marburg, Lassa)	Ebola GP	Not degradation	No	[24]
KSHV	K5	Cell surface downregulation/lysosomal degradation	?	[57]

### BST-2 Domains Important for Restricting Virus Release

To date, the majority of BST-2 mapping studies have revealed species-specific residues important for virus-mediated antagonism of BST-2, but not for the anti-viral function of BST-2. The original studies identifying BST-2 as a viral release restriction factor suggested that the COOH-terminal GPI anchor is necessary for the anti-viral function of human BST-2, as an NH2-terminally hemagglutinin (HA)-tagged mutant missing the GPI anchor and downstream sequences was unable to restrict HIV release [4]. This same group later showed that along with the GPI anchor, both the TM domain and the predicted internal coiled-coil (CC) domain are also important for the BST-2 tethering function [19]. Surprisingly, they discovered that the amino acid sequence of these domains was not important for tethering function. A molecule consisting of structurally similar domains from three unrelated proteins (TM from transferrin receptor, CC from dystrophia myotonica protein kinase [DMPK], and GPI anchor from urokinase plasminogen activator receptor) was able to restrict viral release as efficiently as BST-2. To investigate whether dimer formation plays a role in the BST-2 release function, Andrew et al. and Perez-Caballero et al. both constructed mutants substituted for the putative disulfide-linked cysteines located within the BST-2 extracellular domain. Each group found that when all three extracellular cysteine residues were mutated (C53A, C63A, and C91A), both dimer formation and BST-2 function were prevented, while single and double substitutions had no effect [19], [25], suggesting that promiscuous dimer formation is important for BST-2 anti-viral activity. Conversely, both groups made substitutions for the two putative N-linked glycosylation sites (N65 and N92) and obtained conflicting results. Andrew et al. found that substituting both of these Asn residues with Gln affected glycosylation, but they had no impact upon either BST-2 function or sensitivity to Vpu [25]. In contrast, Perez-Caballero et al. replaced both Asn residues with Ala, which resulted in a non-functional BST-2 [19]. However, because this latter mutant was not efficiently expressed at the cell surface, it is likely that in addition to affecting BST-2 glycosylation, this particular mutation impacted intracellular transport. In summary, the sequence requirements for BST-2 tethering seem to be extraordinarily flexible as long as overall topology and intracellular transport are maintained.

### Molecular Mechanisms of Vpu-Dependent BST-2 Antagonism

Earlier attempts to map the Vpu domains necessary for enhanced virus release were inconclusive. One group found that the two phosphorylation sites within the Vpu C-terminus that are essential for binding to βTrCP were dispensable for virus release [26], while another group showed an approximate 50% reduction in virus release when substitutions were made for these serines [27], [28]. A role for the Vpu TM domain in viral egress was first noted when a Vpu TM mutant that was functional with respect to CD4 downregulation failed to enhance viral release [29]. While all of these studies were performed prior to the discovery that BST-2 inhibits viral egress, recent studies (detailed below) have confirmed a role for Vpu's βTrCP-binding and TM domains in counteracting BST-2.

### The βTrCP-Binding Domain of Vpu Is Important for BST-2 Antagonism

Our group and others have determined that the βTrCP-binding site located within the Vpu cytoplasmic domain is necessary for the downregulation of BST-2 [30]–[34] (Figure 1B). This was demonstrated by showing that a Vpu βTrCP-binding mutant did not induce downregulation or degradation of BST-2. In addition, both a dominant negative βTrCP mutant and an siRNA directed against βTrCP effectively block Vpu's ability to downregulate BST-2. While the βTrCP-binding domain is necessary for counteracting BST-2, it does not appear to be necessary for direct interaction between the proteins, as both wild-type Vpu and the βTrCP-binding mutant co-immunoprecipitate and co-localize with BST-2 [30], [33]. These results suggested that another region(s) within Vpu mediates BST-2 binding.

### The Vpu Transmembrane Domain May Mediate BST-2 Binding

One candidate region for a putative BST-2 binding site is the Vpu TM domain (Figure 1B). While recent data suggest that the Vpu TM domain interacts with BST-2 [32], [35] and is important for Vpu's ability to downregulate BST-2 [5], no comprehensive mapping has been reported thus far.

### The Transmembrane Proximal Region Affects the Subcellular Localization of Vpu

Varthakavi et al. have suggested that the localization of Vpu to a specific pericentriolar compartment of the TGN is necessary for its ability to enhance virion release [36]. The domain responsible for this TGN localization was later mapped to the Vpu TM proximal region, which contains two overlapping putative sorting signals (tyrosine-based YXXΦ and di-leucine based (D/E)XXXL(L/I)) [37] (Figure 1B). This region was first identified in Vpu C, where it was shown to be involved in both the plasma membrane localization of Vpu C and viral egress [38]. Mutagenesis of this region in Vpu B was also shown to reduce viral release [37].

### Degradation of BST-2 in the Presence of Vpu

**Figure 2:**
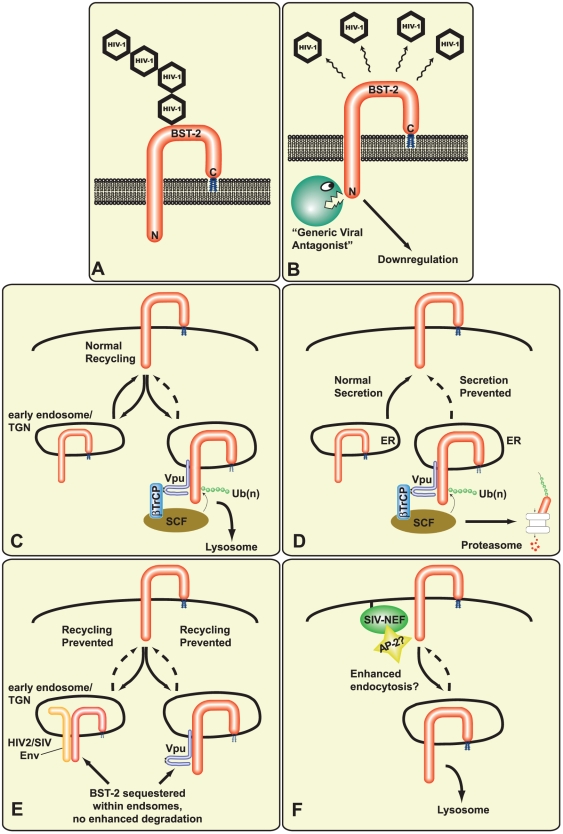
Potential mechanistic models of BST-2 tethering and viral antagonists against BST-2. (A) BST-2 acting as a virion tether in the absence of any antagonist; (B) efficient virus release when BST-2 function is inhibited by a generic viral antagonist; (C) Vpu-mediated βTrCP-dependent degradation of BST-2 via the endosome/lysosome pathway; (D) Vpu-mediated βTrCP-dependent degradation of BST-2 via the ubiquitin/proteasome pathway; (E) HIV-2/SIV_AGM_ Env- or Vpu-mediated BST-2 sequestration; (F) SIV Nef-mediated BST-2 downregulation. Ub(n), mono- or poly-ubiquitin.

Flow cytometry analyses from many studies clearly indicate that the levels of endogenous BST-2 at the cell surface of HeLa cells are markedly diminished in the presence of Vpu [5], [23], [30], [31], [34]. This decrease in cell surface expression could be caused by either BST-2 degradation or BST-2 sequestration within an intracellular compartment (see Figure 2). Due to conflicting data that has likely arisen from the different methodologies employed (see Table 2), distinguishing between these mechanisms has not been straightforward. However, immunoblot analyses from the majority of studies have demonstrated a decrease in total cellular BST-2 levels in the presence of Vpu, which would favor a degradation mechanism [3], [23], [30]–[33]. Table 3 provides a compilation of the reagents and techniques used, as well as the results obtained from each of these mechanistic studies.

**Table 2:** Advantages and Disadvantages of Various Expression and Assay Systems.

BST-2 Expression Systems	Advantages	Disadvantages
Endogenous	• Physiologically relevant• 100% of cells express protein• No need for expression vectors• Correct modifications and localization	• Cell-type limitations (transfection efficiency and viral vector compatibility)• Cannot make mutants• No isogenic negative control
Lenti/retrovirus	• 100% of cells express protein• Isogenic negative control• Easy mutant analysis• Single copy per genome• Expression levels and processing similar to endogenous	• More time consuming to generate stable cell lines• Potential loss of BST-2 expression over time
Transient transfection	• Quick• Isogenic negative control• Easy mutant analysis	• Over-expression anomalies• (processing/secretion defects)• Variable transfection efficiency (cell-type dependent)• Must perform dose-response

BST-2 Molecules	Advantages	Disadvantages
Epitope-tagged	• Well-characterized, efficient antibodies to tag	• Could adversely affect processing, localization and function
Untagged	• Physiological	• Need efficient antibodies to molecule

Viral Egress Assays	Advantages	Disadvantages
Secreted p24/intracellular p24	• Quick• Only measures viral release	• Measures all particle release including non-infectious• Quantitation of immunoblots
HIV reporter assay	• Only measures infectious particles• Reliable quantitation	• Viral entry, reverse transcription, and integration must not be affected
Long-term viral replication	• Most physiologically relevant	• 7–10 days• Allows cell to cell spread, which can mask egress

**Table 3:** Methods and Results from Mechanistic Studies Evaluating Vpu-Mediated Downregulation of BST-2.

Reference	Cell Type	BST-2 Source	Vpu Source	Surface BST-2	Total BST-2	βTrCP-Dependent BST-2 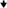	βTrCP-Dependent Egress	VpuTM-Dependent BST-2 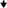	VpuTM-Dependent Egress	Vpu/BST-2 Co-IP	BST-2 Half-Life	Drug Inhibition of Vpu Function	Conclusion
[3]	HeLa	Endogenous	Ad-Vpu	*n.d.*	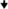	*n.d.*	*n.d.*	*n.d.*	*n.d.*	*n.d.*	*n.d.*	*n.d.*	• Vpu targets BST-2 for degradation
[5]	HeLa	Endogenous	pVphu (transfect)HIV-1pVpu::GFP (subtype B)pVpu::GFP (subtype C)	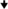 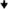 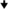 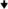	*n.d.* *n.d.* *n.d.* *n.d.*	*n.d.*Yes*n.d.* *n.d.*	*n.d.*Yes*n.d.* *n.d.*	*n.d.*Yes*n.d.* *n.d.*	*n.d.*Yes*n.d.* *n.d.*	*n.d.* *n.d.* *n.d.* *n.d.*	*n.d.* *n.d.* *n.d.* *n.d.*	*n.d.*5 h MG132 = no effect*n.d.n.d.*	• Vpu downregulates surface BST-2• Vpu dependent BST-2 degradation is proteasome dependent• Vpu-βTrCP binding is required• Vpu TM domain is involved
[34]	HeLa	Endogenous	pVphu (transfect)	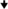	No Δ	Yes	Yes	*n.d.*	*n.d.*	*n.d.*	*n.d.*	Bafilomycin and MG132 (14 h)	• Vpu targets BST-2 between the endosome and lysosome• Lysosomal degradation
[31]	TZM-bl (HeLa)A3.01 (T cell)293T	EndogenousEndogenousExogenous, NH2-term. HA-tag	HIV-1HIV-1pVphu (transfect)	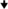 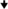 *n.d.*	*n.d.* *n.d. 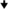 *	*n.d.* *n.d.*Yes	*n.d.* *n.d.*Yes	*n.d.* *n.d.* *n.d.*	*n.d.* *n.d.* *n.d.*	*n.d.* *n.d.* *n.d.*	*n.d.* *n.d.*∼1–3 h, “biphasic”	*n.d.* *n.d.*MG132 and clastolactocystin increase to <18 h	• Vpu-βTrCP dependent BST-2 downregulation• BST-2 degradation is proteosomal
[23]	HeLaMDMCEMx174 (B/T cell hybrid)H9	EndogenousEndogenousEndogenousEndogenous	pVphu (transfect)HIV-1 AdaHIV-1 (long term)HIV-1 (long term)	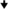 *n.d.*No ΔNo Δ	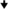 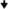 No ΔNo Δ	*n.d.* *n.d.*N/AN/A	*n.d.* *n.d.*NoNo	*n.d.* *n.d.*N/AN/A	*n.d.* *n.d.*YesYes	*n.d.* *n.d.* *n.d.* *n.d.*	*n.d.* *n.d.* *n.d.* *n.d.*	*n.d.* *n.d.* *n.d.* *n.d.*	• Vpu leads to BST-2 degradation in some, but not all, cell types• Viral release may not depend on BST-2 degradation
[30]	HeLaCEM::GFP (T cell)	EndogenousEndogenous	Adeno-VpuHIV-1	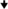 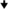	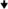 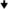	YesYes	Yes*n.d.*	*n.d.* *n.d.*	*n.d.* *n.d.*	Yes*n.d.*	>24 h, Vpu- ∼12 h, Vpu+*n.d.*	CMA inhibits turnover, MG132 does not*n.d.*	• βTrCP-dependent, lysosomal BST-2 degradation
[33]	293T	Exogenous, NH2-term. HA-tag	pVphu (transfect)	*n.d.*	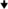	Yes	Yes	*n.d.*	*n.d.*	Yes	*n.d.*	12 h MG132	• βTrCP-dependent, proteasomal BST-2 degradation
[32]	293TCOS7	ExogenousExogenous, FLAG, and MYC tags	pCA-Vpu-RRE+pCa-REVpCA-Vpu-RRE+pCa-REV	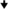 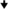	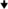 *n.d.*	*n.d.* *n.d.*	*n.d.* *n.d.*	*n.d.* *n.d.*	Yes*n.d.*	Yes*n.d.*	*n.d.* *n.d.*	MG132 stabilizes BST-2 regardless of VpuLysosomal inhibitors cause Vpu and BST-2 to co-localize to the lysosome	• βTrCP-dependent lysosomal BST-2 degradation• Vpu acts upon BST-2 at the plasma membrane• Vpu binds to BST-2 via their TM domains

N/A, not applicable; n.d., not done; 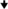, downregulation.

### Vpu-Mediated Degradation Pathways of BST-2

While it remains to be determined whether BST-2 is directly ubiquitinated upon interaction with Vpu and βTrCP, support for a ubiquitin-dependent mechanism was provided by experiments in which the Vpu-mediated downregulation of BST-2 was significantly inhibited by concanamycin A [30], bafilomycin A1 [34], and long-term MG132 treatment (≥12 h) [31], [33], [34]. Concanamycin A and bafilomycin A1 are both vacuolar H(+)-ATPase inhibitors that block endosomal maturation and thus lysosomal degradation. In contrast, MG132 is a proteasome inhibitor that, when used for extended periods, prevents cellular ubiquitin recycling. Since the resulting ubiquitin depletion can affect ubiquitin-mediated endocytosis and other ubiquitin-dependent pathways, MG132-treatment does not necessarily implicate proteasomal degradation. Depending on the drugs used, opposing conclusions have been reached, in which Vpu-mediated degradation of BST-2 occurs via either the lysosome [30], [32], [34] or the proteasome [31], [33] (see Table 3 and Figure 2). Another possible cause for these conflicting results may be the BST-2 expression systems utilized (see Table 2). In general, data supporting a lysosomal degradation mechanism have come from studies of endogenously expressed BST-2, while data supporting a proteasome-dependent pathway have arisen from the use of exogenously expressed, epitope-tagged BST-2, which often results in the accumulation of immature BST-2 within the endoplasmic reticulum (ER) [25].

To further investigate a role for ubiquitination of BST-2, two groups have mutated the cytoplasmic lysine residues of BST-2, which are the most likely targets for ubiquitin addition (Figure 1B). Both groups found that the double lysine mutant retained the ability to restrict viral egress and was still downregulated by Vpu. These data suggest that if BST-2 ubiquitination is required for its viral restriction function or necessary for Vpu-mediated downregulation, then residues other than the cytoplasmic lysines must be the ubiquitin target [33], [34]. A definitive mechanism for the Vpu-mediated degradation of BST-2 awaits a more extensive analysis of the role that ubiquitin plays in this process.

### The Role of BST-2 Endocytosis in the Vpu-Mediated Downregulation of BST-2

Mitchell et al. presented data that indicates a role for the endosomal adapter protein complex member AP-2 (μ2) in the Vpu-dependent downregulation of BST-2 [34]. However, Vpu did not appear to enhance the rate of BST-2 internalization, leading the authors to conclude that Vpu acts after BST-2 is naturally endocytosed. In contrast, Iwabu et al. mutated a dual-tyrosine site in BST-2 (Y6/8A) (Figure 1B) involved in clathrin-dependent endocytosis and found that Vpu was still able to induce BST-2 downregulation, suggesting that natural BST-2 endocytosis is not required for this process [32]. The interpretation of any effects Vpu might have on BST-2 endocytosis are complicated by the conflicting reports regarding which AP-2 subunit, either μ2 [11] or α-adaptin [9], is involved in the natural BST-2 endocytosis pathway.

## Species-Specific Lentiviral Countermeasures against BST-2

The retroviral restriction factor TRIM5α was identified in studies designed to identify host factors responsible for HIV-1 restriction in Old World monkeys [39]. A number of recent publications (described below) suggest a similar species specificity in the abilities of primate lentiviruses to overcome BST-2 restriction by their respective hosts.

### Non-Human BST-2 Proteins Restrict HIV-1

Several studies have found that HIV-1 egress is inhibited by BST-2 proteins from a wide selection of mammalian species. This list includes Old World monkeys, such as rhesus macaques [40], [41], African green monkeys (AGMs) [40], [42], [43], and Mustached monkeys [44], as well as both mice and rats [31], [40]. Thus far, the only primate BST-2 shown not to restrict HIV-1 was found in a species of New World owl monkey (*Aotus lemurinus griseimembra*) [45]. However, when the sequence of this defective BST-2 was compared to that of closely related owl monkeys encoding functional BST-2 proteins, the defect mapped to residue 181 (I 181 T) within the predicted COOH-terminal GPI-anchor signal peptide. This mutation altered normal BST-2 glycosylation, which leads to the inactivation or mistargeting of the protein in this owl monkey species. Taken together, these data suggest that as long as BST-2 is able to mature properly, BST-2 restriction of HIV-1 is remarkably species independent. This generalization was extended further by Sato et al., who showed that when transfected into a variety of mammalian and bird cell lines, human BST-2 can still restrict HIV-1 release. This suggests that BST-2 function requires no species-specific cofactors [46].

### HIV-1 Vpu Does Not Counteract Non-Hominid BST-2

Another interesting aspect of the aforementioned studies was the consistent observation that HIV-1 Vpu counteracts human and chimpanzee (cpz) BST-2, but not BST-2 proteins encoded by non-hominids [31], [40]–[45]. These findings explain the previous observation that regardless of Vpu expression, COS-7 cells (derived from AGMs) tethered HIV-1 upon IFN induction [47]. Further support for the species specificity of Vpu-mediated antagonism of BST-2 came from studies demonstrating that Vpu encoded by SIVmus (which infects Moustached monkeys [*Cercopithecus cephus*]) could antagonize the *C. cephus* and the closely related AGM BST-2s, but not human BST-2. This phenomenon has recently been extended to include numerous other Vpu-expressing simian immunodeficiency virus (SIV) isolates (SIVgsn/mus/mon) [48], [49]. Surprisingly, the Vpus encoded by SIVcpz and SIVgor were inactive against all BST-2s tested. Instead, these SIV strains appear to utilize Nef for this purpose (see below) [48], [49]. Thus, while BST-2's ability to restrict viral egress appears to be pleiotropic, there appears to be a clear adaptation of viral Vpu proteins to their respective host species, with the notable exception of SIVcpz/gor.

### BST-2 Domains Required for Sensitivity to Vpu

**Figure 3:**
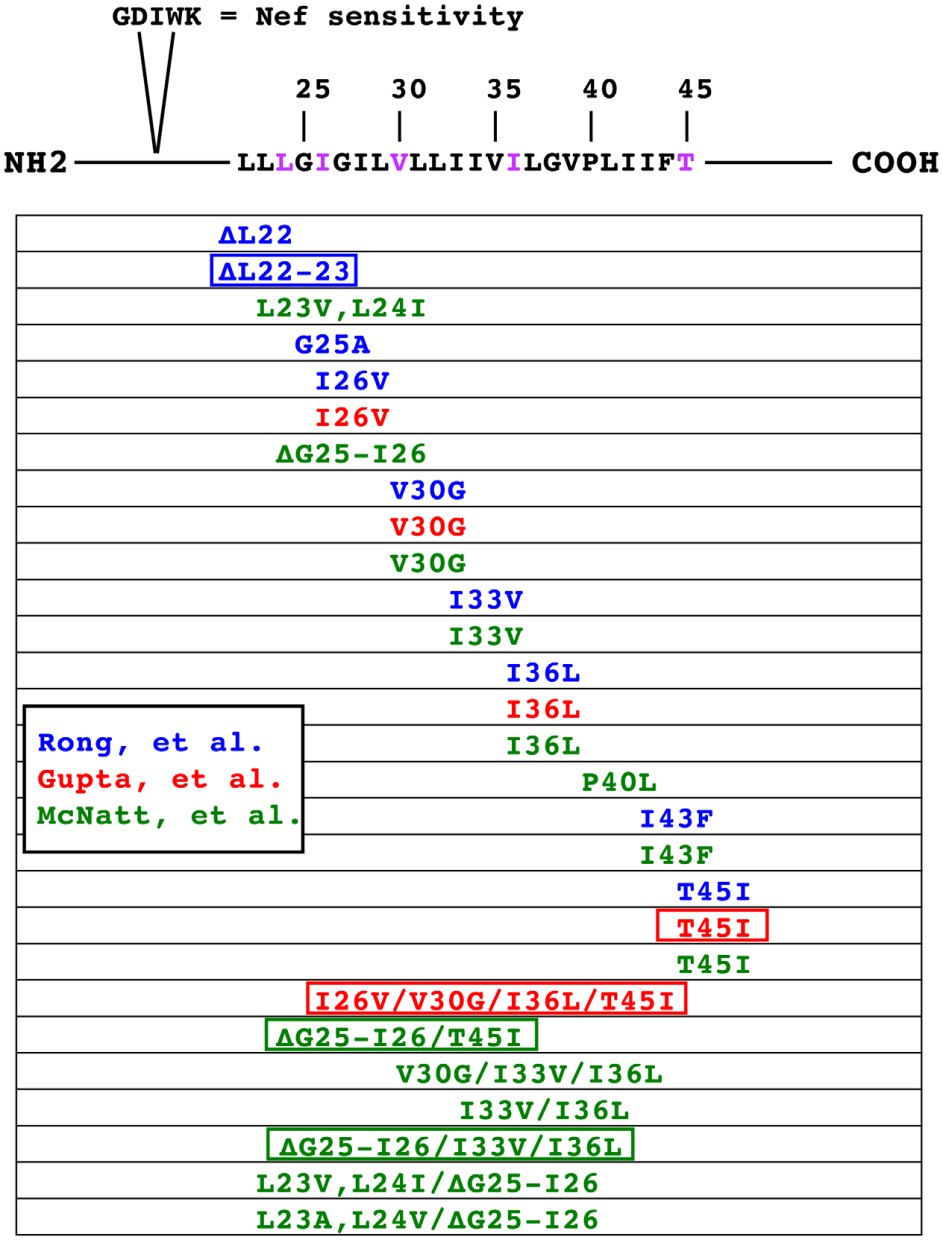
Compilation of BST-2 TM mutants evaluated for sensitivity to Vpu antagonism. The schematic at the top represents human BST-2 with the sequence of the TM domain. Also shown are the five amino acids present in rhesus, but absent from human BST-2, that confer sensitivity to SIV Nef. Residues in purple have been shown to be under positive selection [42]. Three laboratories have generated substitution mutants in human BST-2 that replace the human residue for the corresponding rhesus or AGM residue. Mutants made by each group are color-coded; Rong et al. in blue [43], Gupta et al. in red [42], and McNatt et al. in green [40]. All three groups evaluated their mutants in similar infectious virus-release assays that compared the egress of wild-type HIV-1 to that of ΔVpu HIV-1. Mutants shown in boxes were identified in their respective publications as having a significant impact on sensitivity to Vpu with little to no effect on BST-2 tethering function.

The species specificity of BST-2 antagonism has provided the unique opportunity to map residues within human BST-2 that are required for Vpu-mediated downregulation. Swapping the cytoplasmic, TM, and extracellular domains between human and mouse BST-2 showed that important determinants are present in each human domain that are required for Vpu downregulation [31]. Other studies showed that rhesus BST-2 was downregulated by Vpu when the TM was replaced with that of human BST-2 [40]–[43]. Conversely, replacing the TM of human BST-2 with that of rhesus BST-2 rendered the chimeric protein resistant to Vpu. In an alternative approach, a comparison of primate BST-2 nucleotide sequences suggested that the ratios of non-synonymous substitutions (nucleotide changes that affect the protein sequence) to synonymous substitutions were higher in the cytoplasmic and TM domains compared to those in the extracellular domain [40], [42]. Focusing on these regions led to the identification of residues within the TM of human BST-2 that influenced Vpu-mediated downregulation (Figure 3) [40], [42], [43]. However, due to the wide variation in both BST-2 expression and maturation presented in these studies, no clear consensus has emerged.

## Other Viruses Restricted by BST-2 and Their Countermeasures

BST-2 has been shown to inhibit the release of viral or viral-like particles from a variety of enveloped viruses (Table 1 and references therein). Many of these viruses share little or no homology with one another, thus highlighting BST-2's intrinsic anti-viral function. Because viruses co-evolve with their hosts, it was perhaps not surprising to find that HIV-1 encodes a BST-2 countermeasure in the form of Vpu. Therefore, by extension, one might suspect that other viruses have also developed mechanisms to deal with BST-2. The following section explores the manner in which viruses other than HIV-1 antagonize BST-2.

### HIV-2

In contrast to HIV-1, HIV-2 does not encode a Vpu protein. Regardless, some strains of HIV-2 have been shown to exhibit an enhanced release phenotype in Vpu-restrictive cells [50], [51]. Interestingly, this HIV-2 release function maps to the envelope (Env) protein. Attempts to map those residues that contribute to this phenotype have revealed both a single amino acid (T598) within the gp41 ectodomain [52] and a GYXXθ endocytic-sorting motif within the cytoplasmic tail [53]. Although these studies were performed prior to the identification of BST-2, recent data confirms that egress-competent HIV-2 strains can downregulate cell surface BST-2, and that both residue T598 [30], [41] and the GYXXθ motif [54] may be involved in this process. Mutations that prevent envelope processing are also defective for both egress [52] and BST-2 antagonism [54]. Mechanistically, it has been shown that, like Vpu, HIV-2 Env co-immunprecipitates with BST-2 [54]. However, unlike Vpu, no evidence for HIV-2 Env-dependent BST-2 degradation has been shown. In one study, BST-2 was found to accumulate in the TGN in the presence of HIV-2 Env, suggesting that BST-2 sequestration may be the mechanism whereby HIV-2 Env enhances viral egress [54] (see Figure 2). Of note, HIV-2 Env was also able to antagonize rhesus BST-2 [41], suggesting that HIV-2 Env functions in a species-independent manner.

### Simian Immunodeficiency Viruses

Like HIV-2, most SIV strains do not encode a Vpu homolog. However, in contrast to HIV-2, two recent studies have shown that deleting the SIV *env* gene does not significantly inhibit SIVmac release from cells expressing rhesus BST-2 [41], [55]. Instead, these studies revealed that SIV Nef counteracts BST-2. This inhibition appears to be species specific; while Nef proteins from various SIV strains effectively antagonize BST-2 from their respective hosts, they are inactive against human BST-2 [55]. New evidence suggests that this is also the case for SIVgor and SIVcpz even though they express Vpu [48]. Interestingly, both HIV-1 and HIV-2 Nef appear to have lost much of this functionality, as they do not antagonize human BST-2 [41], [55]. However, they have maintained some detectable activity against the rhesus BST-2 [41]. Using chimeras between human and rhesus BST-2, the region necessary for sensitivity to antagonism by SIVmac Nef was mapped to five amino acids (GDIWK) within the cytoplasmic domain of rhesus BST-2, which are missing in human BST-2 [41], [55] (Figure 3). Mutational analyses have shown that both the Nef myristoylation site [41], [55] and cholesterol recognition motif [41] are important for Nef's ability to counteract rhesus BST-2, thus highlighting the importance of Nef membrane localization (Figure 1C). Nef mutations that abolish downregulation of CD4 and CD28, but not MHC-I, also prevented BST-2 antagonism, suggesting potential mechanistic similarities between Nef-mediated downregulation of both CD4 and BST-2 [55] (see Figure 2). However, aside from the observation that SIV Nef induced cell surface downregulation of rhesus BST-2 [41], no other mechanistic studies have been performed to date. Interestingly, the use of Nef to counteract BST-2 may not be universal among SIV strains. One group has found that, like HIV-2, the SIVagmTan Env downregulates cell surface BST-2 in a species-independent manner [56]. However, this study relied exclusively on exogenously expressed SIVagm Env; *env* deletion viruses were not tested, and control experiments to determine Nef's role were not performed. Further complicating these conclusions, Lim et al. observed only a modest antagonism of AGM BST-2 by wild-type SIVagmTan. They hypothesize that this particular SIV strain may not require a BST-2 antagonist because it does not induce a robust IFN response in vivo [44]. More systematic, comparative studies will be necessary to a) confirm which strains of SIV have evolved BST-2 countermeasures and b) clarify the contributions that Vpu, Nef, and/or Env make towards SIV egress.

### Filoviruses

The inhibition of Ebola VLP release provided the first demonstration that BST-2 limits the egress of a non-retrovirus [47]. Kaletsky et al. screened four Ebola proteins that are known to impact viral egress for their ability to overcome BST-2 [24]. Only the glycoprotein (GP) restored VLP release in cells expressing BST-2. In contrast to Vpu, Ebola GP was found to counteract both murine and human BST-2, suggesting a lack of species specificity. While a direct interaction between GP and BST-2 was inferred from their co-localization and co-immunoprecipitation, no degradation or obvious mislocalization of BST-2 was observed, leaving the mechanism of antagonism by GP unresolved.

### Kaposi's Sarcoma–Associated Herpesvirus

Although KSHV is the only DNA virus currently known to counteract BST-2, our studies of KSHV-encoded immunomodulators established the first viral connection for BST-2 [3]. In a proteomics screen for new host targets of the viral TM ubiquitin ligase K5, we observed that BST-2 levels were reduced in the presence of K5 [3]. More recently, we demonstrated that, similar to other K5 targets, BST-2 is ubiquitinated by K5, resulting in ubiquitin-mediated endocytosis and lysosomal destruction [57]. Ubiquitination occurred at lysines in the cytoplasmic domain of BST-2 (Figure 1A) and removal of the two lysines rendered BST-2 resistant to K5. In contrast, lysine-less BST-2 is still degraded by Vpu [33], indicating that either alternative residues act as ubiquitin substrates or BST-2 is not a direct target of ubiquitin ligases in HIV-1-infected cells. Further analyses revealed that upon knockdown of K5, BST-2 reduced the release of KSHV from HeLa cells [57]. While this result indicates that BST-2 interferes with KSHV egress, further studies will be needed to determine how this interference is achieved since, unlike retroviral budding, herpesviral egress occurs by vesicular transport. Nevertheless, these studies indicate that the anti-viral function of BST-2 acts across an exceptionally wide spectrum of viruses.

## Future Directions

Aside from the mechanistic questions regarding both the manner by which BST-2 inhibits viral egress and the means by which various viruses neutralize this activity, still larger questions remain. For example, how important is it for HIV to improve viral release if the virus can easily spread via cell-to-cell fusion? In long-term viral replication cultures, ΔVpu viruses show increased syncytia formation and cell-to-cell spread [16], suggesting that under these conditions, overall viral replication is not decreased, even though particle release is significantly inhibited. Also, since the majority of studies investigating the BST-2 viral restriction and Vpu countermeasures have been performed in cell lines that are not physiological targets of HIV, will the same conclusions be reached when primary CD4+ T cells are evaluated? Regardless, the very existence and current prevalence of Vpu among HIV-1 subtypes points to an evolutionary pressure to maintain this molecule. This raises the possibilities that a) viral release plays a much larger role in vivo, b) that the selection for the maintenance of Vpu is a result of one of its other functions (i.e., CD4 downregulation), and c) that BST-2 has other important anti-viral function(s) in addition to tethering virions. This latter hypothesis is intriguing in light of the study showing that BST-2 activates the ILT7 receptor on pDCs, leading to inhibition of IFN and proinflammatory cytokine production [13]. This result is somewhat counterintuitive, as it suggests that HIV is promoting immune activation. At the same time, if the goal of this activity is the continued recruitment of T cells to sites of infection, then the result of BST-2 downregulation might be expanded to include both enhanced viral egress and dissemination. Further evidence for an alternative BST-2 function(s) comes from the finding that an entirely synthetic, functional tetherin can be assembled from entirely non-BST-2 sequences [19]. If structure trumps sequence regarding tethering, compensatory mutations within BST-2 would easily arise in response to viral countermeasures, such that there would be little cross-species consensus among BST-2 sequences. That this is not true suggests that BST-2 does indeed perform other functions that require sequence fidelity, although these may or may not serve an anti-viral purpose. While a great deal has been accomplished in this emerging field, many loose ends remain, such that it is too early to become “tethered” to any particular model for either BST-2 function or antagonism.
